# Thoracic epidural arteriovenous malformation causing rapidly progressive myelopathy and mimicking an acute transverse myelitis: A case report

**DOI:** 10.1002/ibra.12070

**Published:** 2022-10-26

**Authors:** Hao Yuan, Yu Pi, Hong‐Su Zhou, Chong Wang, Wei Liu, Yong‐Min Niu, Yang Lan, Dong Chen, Shi‐Ran Liu, Shun‐Wu Xiao

**Affiliations:** ^1^ Department of Orthopedics Affiliated Hospital of Zunyi Medical University Zunyi Guizhou China; ^2^ Institute of Neuroscience Kunming Medical University Kunming Yunnan China; ^3^ Department of Anesthesiology South West Medical University Luzhou China; ^4^ Department of Anesthesiology Affiliated Hospital of Zunyi Medical University Zunyi Guizhou China; ^5^ English Department of College of foreign languages Guizhou University Guizhou Guiyang China; ^6^ Department of Sports Rehabilitation Kunming Medical University Yunnan Kunming China; ^7^ Department of Informatics, Faculty of Business, Economics and Informatics University of Zurich Zurich Switzerland; ^8^ Department of Neurosurgery Affiliated Hospital of Zunyi Medical University Zunyi China

**Keywords:** acute transverse myelitis, arteriovenous malformations, epidural space, misdiagnose, spontaneous hemorrhage

## Abstract

Clinical symptoms of spinal arteriovenous malformations (AVMs) combined with acute spontaneous hemorrhage lack specificity, which leads to misdiagnosis and delays treatment. The current study aimed to analyze the causes of misdiagnosis and review the key points of diagnosis and treatment. We presented an extremely rare case of a 25‐year‐old man whose clinical characteristics mimicked acute transverse myelitis, suffering from rapidly and repeatedly progressive myelopathy with a mass. The pathological diagnosis of the mass was AVM; symptom‐based surgical treatment with posterior decompression and the removal of epidural AVMs during the postoperative 12‐month follow‐up period were performed. The manual muscle testing grade score of the proximal and distal muscles in both lower limbs improved from 1 to 5, and the American Spinal Injury Association motor and sensation grade score improved from B to E. In the case of sudden or progressive spinal cord injury of unknown cause and acute spinal cord dysfunction, there might be a misdiagnosis. The key to a differential diagnosis is to take into account AVMs, and spontaneous hemorrhages and hematomas should also be suspected. Angiography and magnetic resonance imaging are very important for the diagnosis of AVM, and we hope to enhance clinicians' understanding of and vigilance for such diseases.

## Graphical abstract

By presenting an extremely rare case of a 25‐year‐old man whose clinical characteristics mimicked acute transverse myelitis, suffering from rapidly and repeatedly progressive myelopathy with a mass, we concluded that in the case of sudden or progressive spinal cord injury of unknown cause and acute spinal cord dysfunction, there might be a misdiagnosis. The key to a differential diagnosis is to take into account arteriovenous malformations (AVMs), and spontaneous hemorrhages and hematomas should also be suspected. Angiography and magnetic resonance imaging are very important for the diagnosis of AVM.

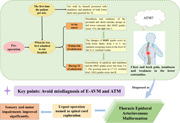

## 1 INTRODUCTION

With the development of advancements in clinical neuroscience and imaging techniques, more knowledge has been gained on vascular malformations. Based on surgical anatomy and the relationship between spatial adjacent and pathological characteristics, diverse and complicated classification methods for spinal vascular malformations have been proposed.^1^ Among these, spinal arteriovenous malformations (AVMs) are classified as extradural, extradural–intradural, and intradural.^2^ Typical epidural arteriovenous malformations (E‐AVMs) originate from the vertebral bodies and extend into the epidural area; a pure E‐AVM is extremely rare, known only through case reports, let alone combined with spontaneous hemorrhage. Thus, its full clinical, radiological, and operative descriptions are comparatively scarce.^3^ What is worse is that it can rapidly aggravate neurological morbidity when there is spontaneous and repeated bleeding. We treated a patient with acute neurologic symptoms caused by nonfunctional arteriovenous hemorrhage more than a year ago, but he was originally misdiagnosed as having acute transverse myelitis (ATM). The main purpose of this paper is to analyze the causes of misdiagnosis from this case and review the key points of diagnosis and treatment to gain increased understanding of these entities in recent decades.

## 2 CASE REPORT

### 2.1 History

**Figure 1:**
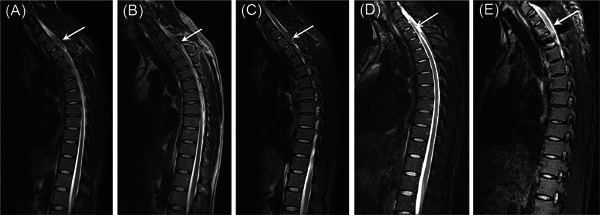
MRI image of the patient. (A, B) Day 7 of symptom onset. Both (A) and (B) show MRI images taken at the time of presentation at another hospital, showing high density of the spinal cord with suspected myelitis 7 days after the onset of the disease. (C) Day 16+ of symptom onset. MRI showed a T1–T2 fusiform mass that was isodense on the sagittal T1‐weighted image and hyperintense on the T2‐weighted image, but hyperintense on the T1‐weighted image in the center of the mass. (D) Day 22+ of symptom onset. Preoperative contrast‐enhanced MRI revealed that there was a significant enhancement, but uneven expansion of the fusiform mass to the level of the C7–T2 vertebral body in the sagittal position. (E) 12 months after surgery. MRI in thoracic vertebra showed decompression of the spinal canal at the level of the C7–T2 vertebra. MRI, magnetic resonance imaging.

A 25‐year‐old man who could not walk by himself presented with numbness and paralysis of both lower limbs that persisted for 7 days, and was treated in his local hospital. Magnetic resonance imaging (MRI) showed hyperintensity in the spinal cord, and myelitis was suspected; hematoma compression was not ruled out (Figure 1A,B). He was eventually diagnosed with acute transverse myelitis (ATM). After 3 days of treatment of dehydration and the trophic nerve, the sensation and movement of his lower limbs returned to normal and he was discharged from the hospital. However, his lower limbs became numb and he was paralyzed again with chest and back pain after 5 days. He was immediately hospitalized, without a significant medical and traumatic history, in our institution on December 29, 2019, except for an upper respiratory tract infection 1 month ago. The patient complained that fatigue before onset of symptoms was obvious, and the exact cause was not clear. General examination: on the first day of admission, his temperature was 36.6°C, pulse was 70 breaths per minute, and blood pressure was 132/88 mmHg. Blood pressure fluctuated between 120–145/70–90 during hospitalization. Physical examination: thoracic and lumbar spine during their lateral pressure were significantly painful, and the muscle tension in both lower limbs was mildly enhanced; neurological examination revealed marked paresthesia and weakness of the proximal and distal muscle groups in the left lower extremity (manual muscle testing [MMT] grade: 4/5). The MMT grade of the right lower extremity was 3, Babinski (+), Oppenheim (+). Other medical examinations indicated no significant changes.

### 2.2 Development of the patient's condition

We initially focused on the treatment of ATM, and managed the patient with mannitol and glucocorticoids; meanwhile, we obtained a sample of the patient's cerebrospinal fluid. The protein count of cerebrospinal fluid (CSF), 1305 mg/l (normal range: 200–400 mg/l), cell count with differential (white blood cell count: 2 × 10^6^/L), glucose, and other parameters were normal. MRI of the cervical, thoracic, and lumbar vertebrae was planned to further characterize the lesion and identify the primary cause for the drastic changes in terms of paralysis in both lower limbs during the 36 h of hospitalization. In this period, the MMT grades were reduced to level 1 and improved gradually to level 3. MRI revealed that the fusiform mass in T1–T2 vertebral bodies was isodense, but hyperintense in the center of the mass on the T1‐weighted image and hyperintense on the T2‐weighted image. The dorsal spinal cord was compressed, and the local subarachnoid space had narrowed, which were suggestive of an epidural space‐occupying lesion in the posterior part of the spinal canal at the level of the T1–T2 vertebral bodies (Figure 1C). We temporarily stopped the emergency surgical intervention taking into account the improvement of symptoms and the preference of the patient and his family. However, again, exacerbation of paralysis and numbness in both lower limbs (MMT grade of the proximal and distal muscles: 1/5) and urination dysfunction were observed 7 days after he was hospitalized. An emergency contrast‐enhanced MRI (compared with the before) revealed that there was significant enhancement, but uneven expansion of the fusiform mass to the level of C7–T2 vertebral bodies, there was an obvious strip‐band enhanced lesion with a clear outline, the compressed dorsal spinal cord had pushed forward, the subarachnoid space was narrow at the level of the T1–T2 vertebrae and the spinal signal was not abnormal (Figure 1D). On the basis of rapidly progressive changes of neurologic deterioration, we diagnosed spinal cord compression. The American Spinal Injury Association (ASIA) motor and sensation score grade was B, and we proceeded with an urgent operation of spinal cord exploration.

### 2.3 Surgical findings

**Figure 2:**
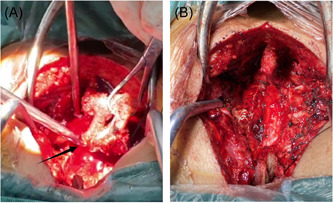
Intraoperative image of focal hemorrhage. (A) The surgical field is blurry due to blood extravasation caused by active hemorrhage in the mass. The arrow indicates the bleeding spot. (B) The surgical field is clear after complete resection of the lesion and thorough hemostasis. [Color figure can be viewed at wileyonlinelibrary.com]

**Figure 3:**
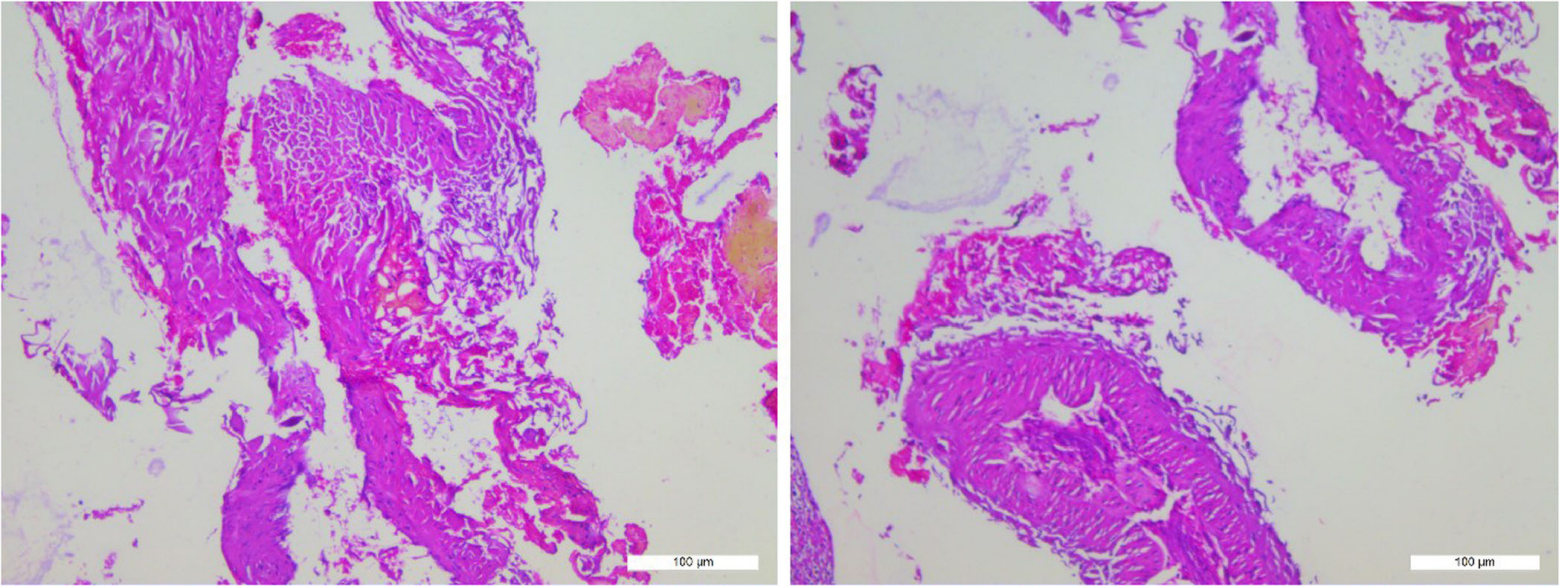
Photomicrograph shows an arteriovenous malformation composed of mixed thin‐ and thick‐walled vascular channels whose lumens are inconsistent and morphology is irregular (H&E stain, ×100). [Color figure can be viewed at wileyonlinelibrary.com]

As soon as the appropriate spinal level had been identified, the surgical strategy involved its posterior exposure. We performed a whole laminectomy of T1–T2 and total extirpation of the mass. During this procedure, we found a mass with an irregular margin located in the epidural space that had no vascular connection to the spinal cord and did not penetrate the dura mater. Surprisingly, the mass seeped outwardly as the pulse beat, which produced a blurry surgery field (Figure 2A), and a dark‐colored small hematoma could be seen at the upper margin of the bleeding area. Various measures (including hematischesis by covering with medical gauze for compression, ligation of malformed vessels, and electrocoagulation) were adopted. Then, the hematoma and the mass were completely removed, followed by patency of compressed vessels (Figure 2B). Finally, to stabilize the corresponding spinal column, a drainage tube was placed and the incision was closed (estimated blood loss: 400 ml). Histopathological examination of the resected mass demonstrated features of an underlying arteriovenous malformation (AVM) (Figure 3), so the final diagnosis of E‐AVM was confirmed.

### 2.4 Postoperative treatments and recovery

**Figure 4:**
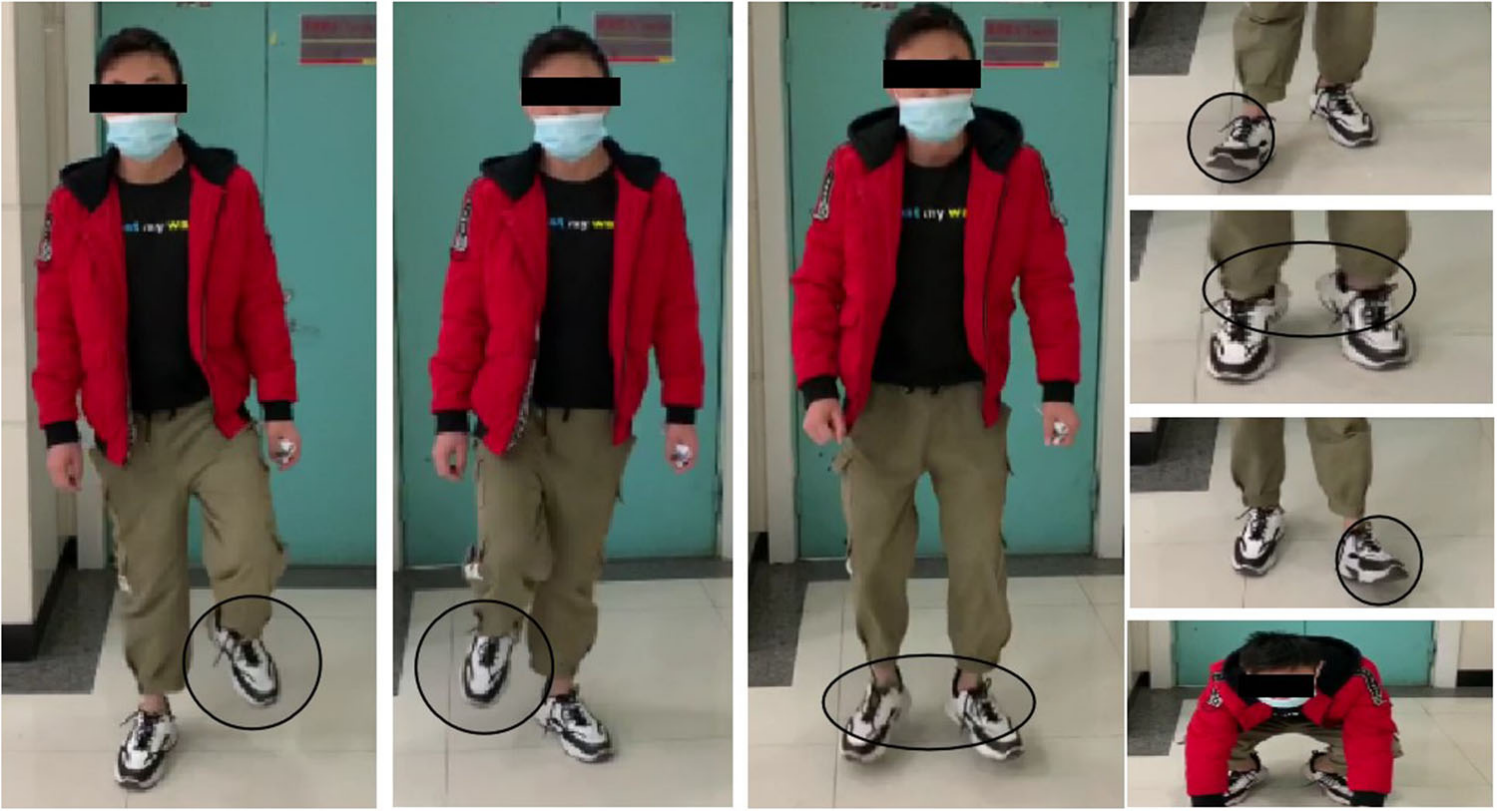
Both lower limb motor nerve functions recovered 12 months after the operation. [Color figure can be viewed at wileyonlinelibrary.com]

After the operation, methylprednisone and ganglioside were used for treatment, and sensorimotor function in the lower extremities gradually recovered. Later, hyperbaric oxygen and rehabilitation therapies were performed. He was able to walk without aid 6 months postoperatively and maintained good walking ability at the final follow‐up visit 12 months postoperatively (Figure 4 and Table 1). Besides, a postoperative MRI performed continuous decompression of c7‐t2 vertebral body corresponding to invasive E‐AVM in the spinal canal (Figure 1E).

**Table 1:** Treatment measures and related improvements in the scores of the patient from the time of admission to our hospital

Progress	Intervening measure	MMT grade score of the lower extremities	ASIA grade score of the lower extremities
Admission	‐	3, 4/5	‐
36 h after admission	Mannitol and glucocorticoids	1→3	‐
7 days after admission	Urgent operation for spinal cord exploration	1/5	B
Postoperation	Methylprednisone and ganglioside	3–4	C
6 months postoperation	Hyperbaric oxygen and rehabilitation therapies	4+	D+
12 months postoperation	Rehabilitation therapies	5	E

Abbreviations: ASIA, American Spinal Injury Association; MMT, manual muscle testing.

## 3 DISCUSSION

In this case, although we experienced difficulty in making a preoperative diagnosis of an E‐AVM, the patient's neurological function returned to normal. The main purpose of the operation is to completely remove the dysfunctional malformed vessels, to prevent palindromia, and decompress the spinal canal. The MMT grade scores of both the lower limbs improved from 1 to 5, and the ASIA motor and sensation grade scores improved from B to E.

**Figure 5:**
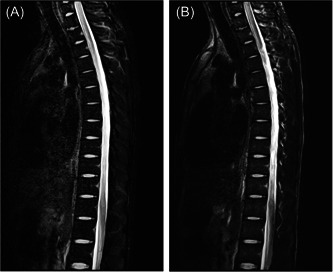
Both (A) and (B) are magnetic resonance imaging of acute transverse myelitis

Symptoms of AVM include sudden pain, sensory disturbances such as weakness and numbness in the extremities, autonomic disorders, bleeding with myelopathy or radiculopathy, and progressive myelopathy when the disease is not hemorrhagic.^4^, ^5^ ATM is a demyelinating disease in which inflammation is confined to the spinal cord and there is predominantly acute spinal cord dysfunction, leading to paralysis, sensory dysfunction (characterized by numbness and paresthesia), and autonomic nerve damage below the lesion level.^6^ Therefore, it is very important to differentiate between the two to avoid misdiagnosis (Table 2). On the basis of the case reported, it was not difficult to identify that the patient had moderate to severe deficits with subacute to rapid deterioration that could have been associated with the following two aspects. First, the typical manifestations were characterized by acute onset of motor, sensory, and urination dysfunction, lacking specificity. Similarly, ATM mostly involves a lesion in the thoracic vertebrae, and not just the symptoms mentioned above; it also often occurs in young individuals with a history of precursor infection or vaccination.^7^ In this case, the patient did have a respiratory infection a month before the onset of symptoms. Second, the neurological function of the patients improved after preoperative treatment, which led us to conclude that the diagnosis of ATM was correct. The patient may not have had much early bleeding from the injury, so it is not obvious on imaging, but compression of the spinal cord causes deformation of the spinal cord, which can lead to an early imaging findings similar to ATM (Video 1). Clinical knowledge of hemorrhage involving in E‐AVMs were not enough, which was the main reason for the misdiagnosis of ATM. However, on reviewing the whole medical history, MR images from the case were quite inconsistent with ATM. Typically, the MRI of ATM is normal or diversified performances including thickening, swelling, unclear boundaries, diffuse hyperintense of a or more lesions on T2‐weighted image (Figure 5).^8^ In addition, the laboratory tests of CSF show that the white blood cell counts usually increase with an increase in CSF protein. Clinically, an elevated CSF protein concentration is also associated with spinal cord tumors, paraneoplastic myelopathies, syringomyelia with spinal block, and spinal cord trauma.^9^ Thus, the diagnosis of ATM is less convincing. Generally speaking, for sudden or progressive spinal cord injury of unknown cause, there might be a misdiagnosis. Meningioma, lymphoma, metastases, hemorrhagic vascular mass, epidural abscess, or other inflammatory conditions should also be considered.

**Table 2:** Comparison of E‐AVM and ATM

Characteristic	E‐AVM	ATM
Pathophysiology	Compression, vascular steal, hemorrhage	Infection
Presentation	Pain, intraspinal hemorrhage, hematoma, limb numbness and progressive myelopathy, neuropathy	Sensory symptoms (numbness, paresthesia, hyperesthesia), weakness, pain; Bowel and bladder dysfunction
Imaging manifestations	MRI showed empty vascular shadows with irregular patches of short T1 hyperintensity in bleeding	MRI showed edema and thickening of spinal cord segments, patchy long T1 and long T2 signals in the lesion
Diagnostic modality	MRI, angiography	MRI, spinal puncture

Abbreviations: ATM, acute transverse myelitis; E‐AVM, epidural arteriovenous malformations; MRI, magnetic resonance imaging.

Put it another perspective, presumably, early remission of neurological deficits maybe because treating and improving slightly damaged nerves causing the mass effect and (or) epidural hematoma without continuously malignant development, the mass effect could exert on the spinal cord by the expanding extradural varix.^10^ There is another possibility of spontaneous resolution of spinal epidural hematoma without surgery, especially in patients with a history of rapid neurological deterioration that is followed by early clinical recovery (such as the patient in our institute), and their radiological studies confirm resolution of the lesion.^11^ When severe symptoms of spinal cord compression occur, the author believes that continuous and repeated bleeding from the malformed arteries and veins cannot be prevented and controlled in time, which plays an important role. Actually, the spontaneous hemorrhage‐restricted epidural is extremely rare and accounts for less than 1% of all spinal epidural lesions.^12^ Coagulopathies, hypertension, increased venous pressure, and vascular malformations are the most common causes. It is also mentioned in the literature that whatever the potential inducing factors, anything that can increase the tension of the malformed vessels, increase arterial blood pressure suddenly, or block venous reflux might lead to spontaneous hemorrhage.^13^ Spontaneous epidural hemorrhages and hematomas are often regarded as venous in origin; yet, we found that in this patient, the mass was bleeding accompanying with the rhythm of the pulse during the operation, indicating that arterial bleeding is considered to be a more likely source for AVM. Thus, arterial spontaneous hemorrhage from spinal E‐AVM should also be considered. However, to diagnose AVM, it is important to rely on radiologic methods.

As we all know, angiography is the gold standard for the diagnosis of almost all vascular diseases, and E‐AVM is no exception. Despite this, the discovery of E‐AVMs is a mostly acute hematoma or hemorrhage, and the main treatment method is emergency surgical intervention, which leads to few opportunities for preoperative angiography, and there are scattered reports of angiographic confirmation of an arteriovenous malformation resulting in an epidural hematoma.^14^, ^15^, ^16^ Spinal angiography has the advantages of defining the presence of deformed vessels, showing the precise location and extent, as well as the arterial source, and the venous exit, even demonstrating vascular malformations in the epidural space angiographically, but not found on MR imaging.^14^ Yet, it is not appropriate for repeated follow‐up and cannot show spinal cord lesions, especially in the case of occult myelangiopathy, indicating that angiography is as equally important as MRI for E‐AVMs. In general, epidural hemangiomas are isodense compared with those of the spinal cord on T1‐weighted images, hyperintense on T2‐weighted images, and brilliantly enhanced with contrast administration. In most cases, hemorrhage and subacute hematoma appearing in the epidural space show a high‐intensity signal on both T1‐ and T2‐weighted images.^17^ However, sometimes, lesions with hemorrhage, hematomas and their liquefaction, or intravascular thrombosis may have different signals on MRI or heterogeneous enhancement.^18^, ^19^ Simply speaking, it is usually difficult to make an accurate diagnosis preoperatively without a typical signal on the MRI study for bleeding nidus in E‐AVM, except for the growing spindle shape (equal with typical manifestation in this case). In our case, there may have also been multiple hemorrhagic events, because the MR images demonstrated untypical signal patterns. In addition, scanning repeatedly MRI is supposed to be considered when being unclear, and surgeons and neuroradiologists must maintain a high index of suspicion for their existence.

E‐AVM, as a nonfunctional vascular mass, is mostly asymptomatic and discovered incidentally, and has to be completely removed to achieve a permanent cure.^20^ If not, it is extremely possible to recruit a new blood supply if only partial excision, and eventually re‐expand to become symptomatic.^21^ For active bleeding leading to the presence of cord compression on MRI, undoubtedly, early operative removal remains the most reliable, definitive therapy. While the ideal surgical outcome is no scathing to useful blood vessels, no interference to the spinal parenchyma, thorough hemostasis, decompression of the spinal canal, and stabilization of the spine. Moreover, combined therapy (hyperbaric oxygen therapy, rehabilitation therapy, and so on), as we mentioned in this case, also plays an irreplaceable role in long‐term recovery.

## 4 CONCLUSION

In summary, spinal AVMs restricted to the epidural space are rare. These lesions can cause severe neurological symptoms, and complete excision of the mass remains a reliable therapy for pure E‐AVMs. For sudden or progressive spinal cord injury of unknown cause and acute spinal cord dysfunction, there may be a misdiagnosis, and the key to a differential diagnosis is to take into account AVMs; spontaneous hemorrhages and hematomas should also be suspected. In addition, surgical intervention, as early as possible, is recommended in the case of rapid and repeated neurological deterioration with spontaneous hemorrhage. Both angiography and MRI are very important for the diagnosis of AVM; if acute compression is not required to deal with surgeons and patients, angiography is the most convictive to find pathogeny. Finally, we hope to enhance the understanding and vigilance of surgeons and neuroradiologists for such diseases.

## AUTHOR CONTRIBUTIONS

All authors made substantial and significant contributions to the conception and design, acquisition, analysis, and interpretation of data, and took part in drafting, revising, and polishing of the article critically for important intellectual content. All authors gave final approval of the version to be published, and have agreed on the journal to which the article has been submitted and to be accountable for all aspects of the work.

## CONFLICT OF INTEREST

The authors declare no conflict of interest.

## ETHICS STATEMENT

The research was approved by the Biomedical Research Ethics Committee of the Affiliated Hospital of Zunyi Medical University (No. KLL‐2020‐275). The authors are accountable for all aspects of the work in ensuring that questions related to the accuracy or integrity of any part of the work are appropriately investigated and resolved. All procedures performed in studies involving human participants were in accordance with the ethical standards of the institutional and/or national research committee(s) and with the Helsinki Declaration (as revised in 2013). Written informed consent was obtained from the patient for the publication of this case report and accompanying images. A copy of the written consent is available for review by the editorial office of this journal.

## Supporting information


**Supporting Information**.

## Data Availability

The data sets used and/or analyzed during the current study are available from the corresponding author on reasonable request.
